# “Knowing Me, Knowing You” the Importance of Networking for Freelancers’ Careers: Examining the Mediating Role of Need for Relatedness Fulfillment and Employability-Enhancing Competencies

**DOI:** 10.3389/fpsyg.2019.02055

**Published:** 2019-09-12

**Authors:** Sofie Jacobs, Ans De Vos, David Stuer, Beatrice I. J. M. Van der Heijden

**Affiliations:** ^1^Antwerp Management School, Antwerp, Belgium; ^2^Department of Management, University of Antwerp, Antwerp, Belgium; ^3^Institute for Management Research, Radboud University, Nijmegen, Netherlands; ^4^School of Management, Open University of the Netherlands, Heerlen, Netherlands; ^5^Department of Marketing, Innovation, and Organisation, Ghent University, Ghent, Belgium; ^6^Hubei Business School, Hubei University, Wuhan, China; ^7^Kingston Business School, Kingston University, London, United Kingdom

**Keywords:** sustainable careers, freelance workers, networking behaviors, employability-enhancing competencies, need for relatedness

## Abstract

Research has shown the importance of engaging in networking behaviors for employees’ career success. Networking behaviors can be seen as a proactive way of creating access to career-related social resources and we argue that this type of proactive career behaviors might be particularly relevant for freelancers who cannot depend on an organizational career system supporting their further development, yet whose careers are characterized by high levels of uncertainty and unpredictability. To date, however, our understanding of how freelancers, being a category of workers that are deprived of an organizational context of support for career development, can safeguard their employability, is limited. Therefore, this study addresses this gap and investigates whether freelancers’ networking behaviors are positively associated with career outcomes, through the mediating role of the need for relatedness fulfillment and employability-enhancing competencies. Hypotheses are tested via Structural Equation Modeling using a sample of 1,874 freelancers from Belgium, France, Germany, the Netherlands, and the United Kingdom. The results generally support our hypotheses, providing evidence for a significant association between networking behaviors and need for relatedness fulfillment, and between networking behaviors and employability-enhancing competencies. Moreover, we found a significant association between need for relatedness fulfillment and employability-enhancing competencies, being the mediators in our research model and the outcomes of career satisfaction and perceived future career opportunities. Implications for career development in the contemporary workplace are discussed, with particular attention for need for relatedness fulfillment, employability-enhancing competencies, and sustainable careers of freelance workers.

## Introduction

One of the most challenging evolutions pertaining to contemporary careers is the shift in the risk distribution regarding career development from the organization to the individual (Guan et al., 2019). Whilst in traditional employment relationships, career management was typically an organizational concern, changes in the socio-economic environment have brought the individual more to the forefront. This implies that, nowadays, the employee is perceived to be the primary actor in career development who is responsible for realizing career success according to his/her personal success criteria (ibid.). Over the past two decades, this has led to a plethora of research addressing individual career management, thereby introducing new career paradigms such as the protean career (Hall, 2004) and the boundaryless career (Arthur et al., 2005).

Yet, most of this research has focused on different categories of workers being employed within an organization (Arthur et al., 2005; Tams and Arthur, 2010). Obviously, this focus is no longer a valid representation of how the world of work is currently evolving, as traditional employer-employee relations are fundamentally changing for a significant group of workers on the labor market (Lo Presti et al., 2018). Indeed, contemporary labor markets are faced with economic volatility and technological changes that have made it more important and, at the same time, also quite challenging for organizations to sustain and further enhance a flexible and highly employable workforce who is able to cope with the ever-increasing speed of changing demands in the world of work. In particular, for independent workers, the above-mentioned changes imply that a career has become less embedded in an organizational setting (ibid.), which means that careers need to be managed more intensely by the person him- or herself, especially when workers get less or no traditional organizational career management support due to their atypical contract type. Moreover, freelancers, as a category of independent workers, have become a key driver for economic performance (Burke and Cowling, 2015), as they can enable or facilitate businesses in an innovation-driven economy (Burke, 2011). As such, nowadays, project work and digital work is getting more important (e.g., Jimenez et al., 2017), which has also resulted into forms of employment where freelancers might be a key in the successful performance of teams.

Whilst career management has typically been regarded from the perspective of the interplay between the organization and the individual, the ‘career playing field’ for independent workers is thus substantially different (Lo Presti et al., 2018), and we argue that, especially for this category of workers, individual career agency is the key to success (Van der Heijden and De Vos, 2015). Accordingly, this article focuses on the social embeddedness of freelancers’ careers, using a sustainable career perspective (ibid.) and, through this, doing justice to the complexity of the connections between individuals and their career-related contexts. A basic premise of sustainable career theory is that, in order to have a sustainable career, a sound interaction of the individual with their surrounding stakeholders is of utmost importance given the increasing complexity, both at work and in one’s private life, and the need to integrate both domains taking a longer-term perspective (De Vos et al., 2019). After all, all stakeholders involved may form important social resources for one’s sustainable career development, however, for freelancers the access to these recourses might be less evident as they are working independent from an organizational context. Therefore, actively engaging in networking behaviors can be an important way to develop and sustain one’s social resources for this category of workers.

In this paper, we address how independent workers’ networking behaviors, seen as a form of personal agency, generate career resources that in turn explain their subjective career success. In particular, we apply a resource management perspective (Hobfoll et al., 2018; Spurk et al., 2019) and investigate the role of two potentially important resources which we expect to operate as mediators in this process, i.e., need for relatedness fulfillment and employability-enhancing competencies. Resources are typically conceived as an important means of helping people obtain personally valued objects or states (Hobfoll et al., 2018) and career success can be regarded as being such a valued state (Spurk et al., 2019). We focus on career resources, which refer to individual resources that reside at the intersection of person-in-environment (Savickas and Porfeli, 2012).

First, through investments in career-related networking, freelancers generate social resources which might fulfill their need for relatedness (Ryan and Deci, 2000), being a basic psychological need that has been found to be an important antecedent of work-related attitudes and behaviors such as, job satisfaction, affective commitment, burnout, engagement, and turnover intentions (Van den Broeck et al., 2016). As the fulfillment of the need for relatedness might be particularly challenging for freelance workers, due to the lack of a network of colleagues, we argue that taking one’s own responsibility or career agency is important in the light of the sustainability of their career (Van der Heijden and De Vos, 2015; De Vos et al., 2019). Second, career-related networking may also enhance freelancers’ employability, which has become a critical resource for sustainable careers in today’s labor markets (van der Heijde and van der Heijden, 2006; Van der Heijden and De Vos, 2015). The unpredictable and rapidly changing context has made it important for all workers to safeguard their career potential by engaging in employability-enhancing activities (De Vos et al., 2011). Given that a career is inherently a relational phenomenon (Hall, 2004), we posit that employability-enhancing competences should be generated through networking behaviors enabling workers to ask for feedback and advice, and to learn from others, to name just a few.

This study contributes to the careers literature and practice in this field in several ways. First, we add new insights into the role of need for relatedness, a basic psychological need (Deci and Ryan, 2000; Van den Broeck et al., 2016), in explaining subjective career success. In particular, our study addresses how individuals who lack embeddedness in an organizational context can take charge of fulfilling their needs for relatedness through engaging in networking behaviors. In doing so, we relate recent insights from the so-called new careers literature, which emphasizes the role of agency in career management (Hall, 2004; Arthur et al., 2005; De Vos and Soens, 2008) with literature on basic psychological needs.

Second, building on earlier empirical research on the added value of employability in the light of career success (van der Heijde and van der Heijden, 2006; Van der Heijden et al., 2009b, 2018; Olson and Shultz, 2013), which has mainly focused on employees who are embedded in organizational settings, we aim to help closing the gap on scholarly knowledge on freelancers. In doing so, we provide important new insights into the careers of an increasingly important group of workers in today’s labor markets. For freelancers, an organizational setting that might create opportunities for employability-enhancing support is missing and hence, they are more dependent on their own initiatives and career agency to make sure that they further develop their competencies in order to protect their career sustainability.

Third, to the best of our knowledge, this is the first empirical work that examines a mediation model between networking behaviors and career outcomes, taking a resource management perspective (Spurk et al., 2019) and, as such, responds to the call for further theory development and strong empirical studies that advance our understanding of sustainable careers (Savickas and Porfeli, 2012; De Vos et al., 2019). Specifically, in this paper, we study the mediating role of need for relatedness fulfillment and employability-enhancing competencies, being the mediators, in the relationship between networking behaviors and career outcomes.

The remainder of this paper is structured as follows. In the next section, we start with a condense overview of literature on the role of networking behaviors in the light of (future) career success, thereby applying a resource management (Spurk et al., 2019) and sustainable career perspective (De Vos et al., 2019) and we discuss the mediating role of need for relatedness fulfillment and employability-enhancing competencies. Next, the methodology of this study is described, followed by its empirical results. Finally, theoretical and practical implications for (research on) career development in the contemporary workplace are discussed, with particular attention for sustainable careers of freelance workers.

## Theory and Hypotheses Development

**FIGURE 1:**
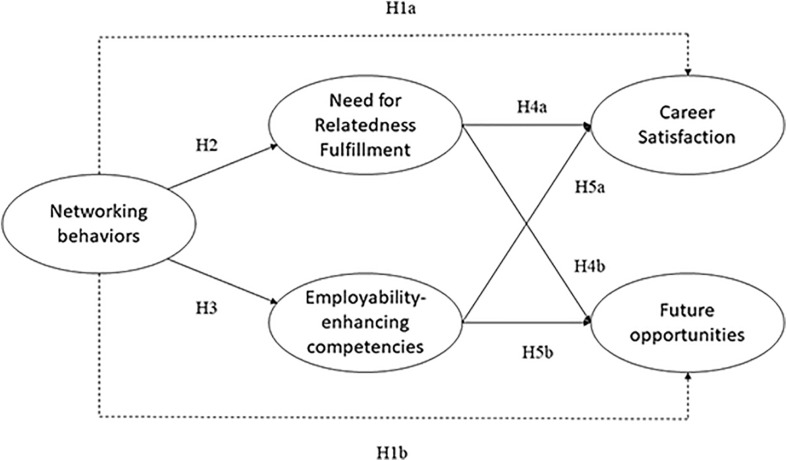
Conceptual model.

We expect that networking, as a form of proactive behavior focused on generating and maintaining career resources, will be positively related to freelancers’ subjective career success (operationalized in terms of career satisfaction and positive career outlook), through the mediating role of need for relatedness fulfillment and employability-enhancing competencies (see Figure 1). This general notion will be unraveled by means of different hypotheses that we will ground theoretically in the following section.

### Importance of Career-Related Networking Behaviors for Subjective Career Success

Subjective career success is typically defined as a worker’s experience and subjective evaluation of achieving personally meaningful career outcomes (Seibert et al., 2001; Ng et al., 2005). As such, subjective career success is most often studied backward looking, with the focal person considering and evaluating the career experiences he or she has encountered to date, in view of personal success criteria. Career satisfaction is one of the most frequently applied measures of subjective career success (Greenhaus et al., 1990). In this manuscript we study freelancers’ general feelings of satisfaction with their career which is most relevant given their independency from traditional organizational career structures (Spurk et al., 2019). In addition, career success can also be considered taking a forward-looking perspective (Eby et al., 2003). A person’s belief to have valuable future career opportunities is particularly relevant in a career context characterized by increased unpredictability (Eby et al., 2003) – a situation which is especially applicable to freelancers’ careers. Therefore, in this paper we also include both general career satisfaction and perceived future career opportunities as indicators of subjective career success.

Career-related networking represents proactive attempts by individuals to develop and maintain personal and professional relationships with others, for the purpose of mutual benefit in their work or career (Forret and Dougherty, 2001). In a boundaryless work environment that is characterized by frequent movement within and across organizations (Arthur and Rousseau, 1996; Sullivan, 1999), and wherein the responsibility for one’s career has shifted from the organization to the individual (Hall, 1996, 2002), forming multiple developmental relationships through career-related networking behaviors has taken on greater emphasis (Higgins, 2000; Higgins and Kram, 2001; de Janasz et al., 2003). As such, nowadays, networking is conceived to be a key human capital skill that is unique in its ability to increase an individual’s social resources (Baker, 2000) (see also social resources theory; Lin et al., 1981a, b; Lin, 1999).

We contend that these social resources can provide individuals with a substantial advantage in their careers and that investing in networking behaviors might be an important means to protect its sustainability. As such, career-related networking can be seen as a form of resource management (Spurk et al., 2019) whereby individuals actively engage in generating social resources through networking. We posit engaging in such behaviors is particularly important for freelancers who, due to the lack of embeddedness within an organizational context, need to find alternatives for organizational career support systems. In particular, within the careers literature, earlier scholarly work supports the idea that career-related networking, which is considered as a core dimension of career self-management, is indeed important for career success (e.g., Abele and Bettina, 2008; De Vos et al., 2009; Wolff and Moser, 2009). For freelancers, these career-related networking behaviors, such as building strong relationships with client organizations and putting effort into developing and maintaining a professional network with other freelancers, can be critical for sustaining and further enhancing their career success and future career prospects (Lo Presti et al., 2018).

In a related sense, a basic premise of sustainable career theory (Van der Heijden and De Vos, 2015; De Vos et al., 2019) is that, in order to have a sustainable career, interaction of the individual with their surrounding stakeholders is important for sustainable career success (ibid.). Consistent with the conceptualization of a sustainable career (De Vos et al., 2019), and applying a stakeholder perspective (cf. Colakoglu et al., 2006) we also underline the possible importance of resources in the person’s context. Typical context resources that have been studied earlier, within the work context, include organizational support for career development (e.g., Barnett and Bradley, 2007), supportive relationships incorporating mentoring (Allen et al., 2004), developmental networks (Bozionelos, 2008), and leadership (Janneck et al., 2012).

Whilst most of our scholarly insight in the prevalence and importance of contextual resources has been obtained from research within organizational settings that are characterized by typical employer-employee relationships, the generalization of earlier knowledge to independent workers cannot be taken for granted. As independent workers’ careers are more boundaryless and are typically developed across a wide range of assignments for different organizations, those contextual resources may be more varied and spread across several organizationally bound social spaces (De Vos and Van der Heijden, 2015). Additionally, extra-organizational resources, such as professional networks or labor market intermediaries, might be important in the light of their career outcomes as well (Arthur et al., 2005). For instance, for independent workers, investing in developing and maintaining strong professional networks with other freelancers (e.g., the rise of co-working spaces), as a means to buffer against the risks of being entirely depending on themselves (van den Born and van Witteloostuijn, 2013) may be an important resource in view of protecting and further enhancing the sustainability of their career.

In this paper, building on the resource management (Spurk et al., 2019) and sustainable career perspectives (De Vos et al., 2019) we propose that networking is important for freelancers’ subjective career success via two pathways, i.e., through fulfilling their basic psychological need for relatedness and through increasing their employability-enhancing competencies. We focus on career-related networking behaviors, whereby strong relationships are developed through frequent contact and emotional investment with important peers (Forret, 2006). We propose that by engaging in such networking behaviors valuable resources can be developed that both fulfill a freelancer’s need for relatedness, and enhance their employability-enhancing competencies.

To conclude, we posit that social resources embedded in career-related networks will provide benefits to individuals engaging in networking behaviors, including greater and more timely access to information, greater access to financial or material resources, and greater visibility, legitimacy, or sponsorship within a social system (Nahapiet and Ghoshal, 1998) which are all important for a successful and sustainable career (Bozionelos, 2003, 2008). This leads us to the formulation of our first, baseline hypothesis about the relationship between career-related networking and career success.

*Hypothesis 1:* Networking behaviors are positively related to freelancers’ subjective career success, operationalized as career satisfaction (H1a) and perceived future career opportunities (H1b).

In what follows, we further specify the mechanisms through which this indirect relationship is expected to occur.

### Relationship Between Networking Behaviors and Need for Relatedness Fulfillment

Self-determination theory (Ryan and Deci, 2000) is built on the premise that people seek to fulfill three basic psychological needs at work: need for autonomy, competence, and relatedness (Ryan and Deci, 2000; Gagné and Vansteenkiste, 2013). When these three types of needs are satisfied, people have been found to be more strongly engaged at work (Van den Broeck et al., 2008), and need satisfaction appears to also be associated with career satisfaction (Shaver and Lacey, 2003). The fulfillment of the need for relatedness might be particularly challenging for freelancers, given that they are not structurally embedded in an organizational setting, yet, often working from home, and lack the context of a team of ‘real’ colleagues at work as they frequently change assignments, are often working on multiple assignments for diverse clients at the same time, and are most often excluded from social activities that companies organize for their employees.

We argue that engaging in networking behaviors can be seen as a proactive strategy to satisfy the basic psychological need for relatedness. As such, the social resources that freelancers generate when engaging in career-related networking behaviors, might help them to satisfy their need to be connected to other people and to build up career-instrumental or socio-emotional network ties (Bozionelos, 2015) (see also Fombrun, 1982; Saint-Charles and Mongeau, 2009). Resources of a career-related social network refer to the benefits that may be derived in terms of valuable career outcomes for the individuals participating in these. In particular, developing relationships with high-status individuals has the potential to provide not only beneficial longer-term outcomes, such as promotions and salary progression (Gould and Penley, 1984; Michael and Yukl, 1993; Forret and Dougherty, 2004), but also more immediate benefits such as information and ideas, social support, job search assistance, and business assistance (e.g., providing business leads and gaining access to financial resources) (Forret et al., 1997). These benefits underscore the importance of relationships to career decision-making and outcomes, as career development is inherently relational (Hall, 1996, 2004). In that perspective, Yip et al. (2018) note that supportive work relationships can have a positive influence on autonomous motivation of employees.

Building on these empirical findings and theoretical arguments, we expect that freelancers can satisfy their need for relatedness through engaging in networking behaviors.

*Hypothesis 2:* Networking behaviors are positively related to freelancers’ need for relatedness fulfillment.

### Relationship Between Networking Behaviors and Employability-Enhancing Competencies

In order to remain employable and to ensure life-long employment and personal career success (De Vos et al., 2011), all kinds of workers, with freelancers being no exception, need to possess the right competences to do their work effectively throughout their career (Van der Heijden and De Vos, 2015). This encompasses that they need to focus on the question ‘what are the right competences?’ on an on-going basis (Wright and Snell, 1998; Rodriguez et al., 2002) and that they not only need to invest in domain-specific expertise, but also need to develop competencies that enable them to be proactive and flexible, to handle ambiguity (both in their private life and at work), and that they need to manage all kinds of multiple tasks, stemming from the need to align all kinds of possibly competing demands in both life spheres (home and work), simultaneously (van der Heijde and van der Heijden, 2006).

Up until now, the value of networking behaviors as a specific strategy for building up employability-enhancing competencies that are vital in the light of one’s career sustainability has been largely neglected in earlier scholarly work (Bozionelos, 2015). We argue that especially for freelancers, who work highly independently and who cannot benefit from organizationally bound career support systems, participating in social networks might have strengthening power for them, in terms of augmenting their knowledge and skill sets (i.e., their employability competencies), herewith enabling them to stay on top of innovations and industry news (Smith, 2010). In support of this, recent empirical work on business students’ networking behaviors (Batistic and Tymon, 2017) demonstrated that networking was positively related to internal and external perceived employability through increased access to resources.

Apart from the professional knowledge that is required to perform a specific job, in order to survive in the contemporary workplace, Defillippi and Arthur (1994) already stressed this issue and referred to the importance of three ‘ways of knowing’: knowing-why, knowing-how, and knowing-whom. The latter, refers to an individual’s set of interpersonal relationships, career-related networks, mentoring and all available contacts that accumulate over time (Arthur et al., 1995; Eby et al., 2003), within and outside one’s current organization. These are expected to provide venues for career support and personal development (Parker and Arthur, 2000), which are necessary ingredients for a so-called intelligent career that is characterized by the capacity to undertake successful career transitions (Arthur et al., 1995) (see also Akkermans et al., 2013).

Employability (or career potential) (van der Heijde and van der Heijden, 2006) lies at the basis of optimal employee functioning (Vanhercke et al., 2014), and is a key characteristic for employees that are required to survive in contemporary labor markets (Fugate et al., 2004; Rothwell and Arnold, 2007; Van der Heijden et al., 2009b; Forrier et al., 2015). Two approaches to employability have been distinguished; input-based approaches (e.g., Fugate et al., 2004; van der Heijde and van der Heijden, 2006), that emphasize personal strengths as a basis for one’s employability, and outcome-based approaches (e.g., Rothwell and Arnold, 2007; De Cuyper and De Witte, 2011), that aim to directly assess the chance of employment (Vanhercke et al., 2014; Forrier et al., 2015). In this empirical study, we are interested in investigating the predictive validity of career-related networking for freelancers’ careers (being the outcome), through employability-enhancing competencies (being the mediator). Therefore, in this contribution, we adopt the competence-based operationalization of employability by van der Heijde and van der Heijden (2006), who defined the concept as the individual’s “capacity of continuously fulfilling, acquiring, or creating work through the optimal use of competences” (p. 453). For freelancers, this conceptualization implies their capacity to acquire and fulfill employment for present or new customer(s), and with regard to future prospects (van der Heijde and van der Heijden, 2006; Van der Heijden et al., 2018). We focus on self-perceptions of the freelancers’ employability-enhancing competencies, based on the idea that these perceptions are the main drivers for their behavior (Katz and Kahn, 1967).

Since employability cannot be seen as a uni-dimensional construct, the authors (van der Heijde and van der Heijden, 2006; Van der Heijden et al., 2018) distinguished between five competence-based employability dimensions. The first focuses on domain-specific occupational expertise (knowledge, skills, including meta-cognitive ones, and social recognition by important key figures), whereas the other four more general dimensions focus on job-related matters and one’s broader career development aspects: anticipation and optimization; personal flexibility; corporate sense; and balance [see also van der Heijde and van der Heijden (2006) for an elaborate overview].

Wolff and Moser (2009) and Spurk et al. (2015) already claimed that networking behavior is a critical factor in career development (see also Thompson, 2005). In line with Smith (2010), we argue that the responsibility to actively participate in networks as a valuable strategy to enhance one’s employability (or career potential) (see also Van der Heijden et al., 2009a; Van der Klink et al., 2014) rests principally on the shoulder of individual workers, in particular in case of freelancers. This leads to the third hypothesis:

*Hypothesis 3:* Networking behaviors are positively related to freelancers’ employability-enhancing competencies.

### The Mediating Role of Need for Relatedness Fulfillment and Employability-Enhancing Competencies

The recent career literature provides evidence for important career-related resources that are necessary to navigate one’s career (Hirschi et al., 2018). Moreover, relevant indicators of career success, such as salary or performing meaningful or fulfilling work, and being engaged in significant interactions with peers at work, can be seen as resources that are valuable in their own right, and that help individuals to attain additional goals in life (Spurk et al., 2019). Yet, resources also refer to the means through which career success can be obtained (Hirschi et al., 2018). Indeed, inherent to the notion of agency in sustainable careers (De Vos et al., 2019) is that workers should not only be actively engaged in affecting their (short-term) career success outcomes, but should also foster and further enhance their employability competencies (or career potential) (van der Heijde and van der Heijden, 2006) through the opportunities they encounter, the choices they make, and the learning cycles they go through while interacting with colleagues through networking (Van der Heijden et al., 2009a; Van der Klink et al., 2014). The individual’s career potential, in turn, influences their subsequent career opportunities and outcomes (Akkermans and Tims, 2017).

As such, a worker’s career potential might benefit from having multiple developmental relationships. These relationships have been shown to be associated with greater work satisfaction, career progress, and retention (Higgins, 2000). Overall, the powerful impact that relationships with others can have on one’s career is recognized (Seibert et al., 2001). Seen from this perspective, our mediation model is built upon the notion that the way in which individuals utilize their resources (Hirschi et al., 2018) is critical for one’s career success.

Thus, in the light of the present study, we hypothesize that employability-enhancing competencies and need for relatedness fulfillment mediate the relationship between networking behaviors and subjective career success. In particular, we assume that career success requires the relatedness to others in one’s career and that it requires a freelancer to possess employability-enhancing competencies that are necessary to meet the demands in their work and careers, two conditions that can be enhanced by engaging in networking behaviors. This leads to Hypotheses 4 and 5:

*Hypothesis 4*: Need for relatedness fulfillment mediates the relationship between freelancers’ networking behaviors and subjective career success, operationalized as career satisfaction (H4a) and perceived future career opportunities (H4b).
*Hypothesis 5:* Employability-enhancing competencies mediate the relationship between freelancers’ networking behaviors and subjective career success, operationalized as career satisfaction (H5a) and perceived future career opportunities (H5b).

## Materials and Methods

A survey research was conducted among a sample of 1,874 freelancers in Belgium, France, Germany, the Netherlands and the United Kingdom. Participants were contacted via an agency specialized in conducting panel surveys. Participation was voluntary and responses were completely anonymous. A written informed consent was obtained from participants before they completed the survey. For this research, we only included those freelancers indicating that they were working full-time as an independent, resulting in a final sample of 1,463 freelancers. This final sample contained 196 responses from Belgium, 395 from France, 246 from England, 334 from Germany and finally 293 from the Netherlands. Furthermore, 26.18% of the respondents were part of a network of freelancers. On average, these individuals were active as independents for 16.34 years (*SD* = 12.56) and had a mean career length of 25.40 years (*SD* = 14.24). Furthermore, 47.3% of our sample were men.

### Measures

*Networking behaviors* were measured using the ‘networking building’ subscale of the proactive career behaviors instrument developed by Strauss et al. (2012) and consisted of five items. A sample item reads “I am building a network of contacts or friendships to provide me with help or advice that will further my work chances.” Some item wordings were slightly adjusted in order to reflect the work context of a freelancers rather than an employee. Reliability was excellent (Cronbach’s α = 0.91).

*Need for relatedness fulfillment* was measured by the five items from the sub-dimension ‘relatedness’ of the work-related basic needs satisfaction scale developed by Van den Broeck et al. (2010). A sample item reads “At work, I feel part of a group.” Reliability was adequate (Cronbach’s α = 0.66).

*Employability-enhancing competencies* were measured using the 22 items of the validated short-form Employability instrument (Van der Heijden et al., 2018; originally developed by van der Heijde and van der Heijden, 2006). When needed, a slight adaptation of item wording was made such that all items referred to the work context of a freelancers instead of a job performed by an employee. This measurement instrument consists of five competence-based dimensions (i.e., occupational expertise: e.g., *During the past year, I was, in general, competent to perform my work accurately and with few mistakes*; anticipation and optimization: e.g., *I take responsibility for maintaining my labor market value*; corporate sense: e.g., *I support the operational processes within the organization(s) I work for*; personal flexibility: e.g., *I adapt to developments within the organization(s) I work for*; and balance: e.g., *My work and private life are evenly balanced*) was used. These scales have been found to be valid in terms of predictive and discriminant validity (also Van der Heijden et al., 2009b; van der Heijden and Bakker, 2011; Veld et al., 2016). The Cronbach’s alphas of the subscales ranged from good (Cronbach’s α = 0.81, personal flexibility) to excellent (Cronbach’s α = 0.92, occupational expertise). These results are very much in line with reliability coefficients found in other research that used these scales (Van der Heijden et al., 2009b). For our model we use these subdimensions as observed variables loading on the superordinate latent dimension of Employability-enhancing competencies. Using this approach, reliability for Employability-enhancing competencies is good (Cronbach’s α = 0.81).

*Career satisfaction* was assessed using three items from Parasuraman et al. (1992). Respondents indicated their level of satisfaction with their career progress and success. An example item is “How satisfied are you with the progress you have made in your career until now?” Responses were given on a five-point scale ranging from 1 (dissatisfied) to 5 (very satisfied). Reliability for this scale was excellent (Cronbach’s α = 0.92).

*Perceived future career opportunities* were measured using three items that assessed respondents’ beliefs in their chances of obtaining new assignments with current and future clients in the future. This scale was based upon the perceived employability scale originally developed by De Cuyper and De Witte (2008). A sample item reads “I am optimistic that I will obtain new assignments or projects from my current clients in the future.” Reliability of the scale was good (Cronbach’s α = 0.83).

### Analytical Strategy

We used structural equation modeling to test our hypotheses. Our theoretical model was tested using the lavaan package (Rosseel et al., 2018) version 0.6.3, using R version 3.5.2. First we tested the measurement model with confirmatory factor analysis for the latent variables. Secondly, we tested our structural model and compared a full mediation model with a partial mediation counterpart. In order to adequately capture global model fit, we used a combination of goodness-of-fit indices, as is appropriate in SEM analyses (Kline, 2015). There is still ongoing debate on what constitutes a good fit SEM and as a consequence, quite a few guidelines exist. The configuration of fit indices chosen for evaluating model fit were CFI, TLI and RMSEA. As a rule of thumb, a CFI and TLI > 0.90 and a RMSEA < 0.08 indicate an adequate fit between the model and the data (Browne and Cudeck, 1993; Hoyle, 1995). Other authors suggest even more stringent criteria, with CFI, TLI > 0.95 and RMSEA < 0.05 as indicators of good fit (Hooper et al., 2008). When comparing mediation models, we used a Chi-square test to test whether to retain a full or partial mediation model. This is appropriate, since these models are nested in one another. We included career length (measured in years) and number of years working as an independent, and a binary variable indicating whether one belongs to a network of independents (0 = not part of a network, 1 = part of a network) as control variables to our model. Furthermore, when estimating indirect effects in our mediation model, we used a Sobel-test, which is seen as a conservative test for indirect effects (Sobel, 1982; Preacher and Leonardelli, 2001).

## Results

### Confirmatory Factor Analysis

First, a general measurement model was constructed. The general measurement model was defined by loading observed variables onto their respective latent variables. This resulted in a model with 5 latent factors and 21 observed variables. Five items loaded on networking behavior, five items on need for relatedness fulfillment, the five sub-factor scores loaded on employability-enhancing competencies, three items on career satisfaction and finally three items loaded on perceived future career opportunities.

We compared this to a single-factor model in order to assess common-source bias (Podsakoff et al., 2003). Fit for the single-factor model was deemed inadequate [χ^2^(189) = 7662.517, *p* < 0.001, CFI = 0.53, TLI = 0.47, RMSEA = 0.170], suggesting that common source bias is an insufficient explanation for results found. Model fit substantially improved over the single factor model for the general model, but not quite adequate [χ^2^(179) = 2218.289, *p* < 0.001, CFI = 0.87, TLI = 0.85, RMSEA = 0.091].

Next, we assessed local fit indicators to explore whether fit could be improved by specifying extra parameters. To do this, we used a combination of theoretical reasoning and modification indices in order to re-specify our model, as is appropriate in SEM (Kline, 2015). Consequently, we dropped two observed variables due to their low loadings (λ < 0.3). The dropped variables were two items from the self-determination scale, namely: “I don’t really feel connected with other people at my job” (λ = −0.17) and “I often feel alone when I am with my colleagues/clients” (λ = −0.16). As a consequence, scale reliability substantially increased (Cronbach’s α = 0.79). It seems that these items do not adequately capture the experience of belongingness according to freelancers.

Finally, after covarying three pairs of items, fit was deemed adequate for the measurement model since both CFI and TLI were above 0.90 and RMSEA was lower than 0.08 [χ^2^(139) = 850.83, *p* < 0.001, CFI = 0.95, TLI = 0.94, RMSEA = 0.061]. The first covaried pair was: “I seek advice from others within my field of work about additional training or experience that I need to improve my professional prospects” and “I engage in conversations with others within my field of work about the training or work I need to develop the skills that will enhance my future job opportunities.” These items refer to actively conversing with others, whilst other variables of this dimension refer to the building of a network (“I am building a network of contacts with others within my field of work on which I can rely”) thus providing a likely explanation for the residual positive correlation. The second pair that was covaried were the constructs of “Occupational expertise” and “Personal flexibility,” which had a residual positive correlation. These two bear some more resemblance to each other than the other constructs in terms of employability-enhancing competencies, explaining the residual correlation. The last covaried pair was between “Balance” and “Corporate sense,” which had a negative residual correlation. This probably represents the fact that part of the employability-enhancing competencies dimension also refers to the management of paradoxes. To a certain extent, these two can also be construed as opposing goals, and this becomes apparent when taking into account the superordinate latent construct of Employability-enhancing competencies.

Since our sample was taken from multiple countries we also checked for metric invariance of the scales. Partial metric invariance is a minimal assumption that needs to be satisfied in order to build an adequate statistical model (Kline, 2015). Before checking for metric invariance, we checked configural invariance, which was found to be adequate [χ^2^(695) = 1858.85, CFI = 0.93, TLI = 0.91, RMSEA = 0.078]. Consequently, metric invariance was tested and we also found model fit to be adequate [χ^2^(751) = 1973.25, CFI = 0.92, TLI = 0.91, RMSEA = 0.077].

At this point we extracted a correlation matrix in order to provide a preliminary exploration of our hypotheses and also included basic descriptive statistics of our variables (Table 1). In line with Hypothesis 1, there is a high positive correlation between Networking Behaviors and Career Satisfaction. We also found some preliminary support for Hypotheses 2 and 3 as indicated by the positive correlations between Networking Behaviors and Need for Relatedness Fulfillment on the one hand and Networking Behaviors and Employability-Enhancing Competencies. Furthermore we also find positive relations between Need for Relatedness and Employability-Enhancing Competencies and both indicators of Career Success, namely Future Opportunities and Career Satisfaction, providing a small indication that Hypothesis 4 and 5 hold water.

**TABLE 1:** Basic descriptive statistics, correlation matrix of latent variables, Cronbach’s alpha on the diagonal.

	**Mean**	***SD***	**NB**	**NFR**	**PE**	**CS**	**FO**
Networking Behavior (NB)	3.28	0.99	**0.91**	–	–	–	–
Need for relatedness fulfillment (NFR)	3.84	0.60	0.544	**0.79^∗^**	–	–	–
Perceived employability (PE)	4.24	0.77	0.506	0.587	**0.81**	–	–
Career Satisfaction (CS)	3.89	0.94	0.280	0.380	0.676	**0.92**	–
Future Opportunities (FO)	3.72	0.84	0.416	0.478	0.687	0.637	**0.83**

*All correlations are significant at the *p* < 0.001 level. ^∗^Note that Cronbach’s alpha is higher for NFR than reported in the methods, this is due to the fact that we dropped items due to their low loading on the construct. Bold values represent Cronbach’s alpha.*

### Structural Model

**FIGURE 2:**
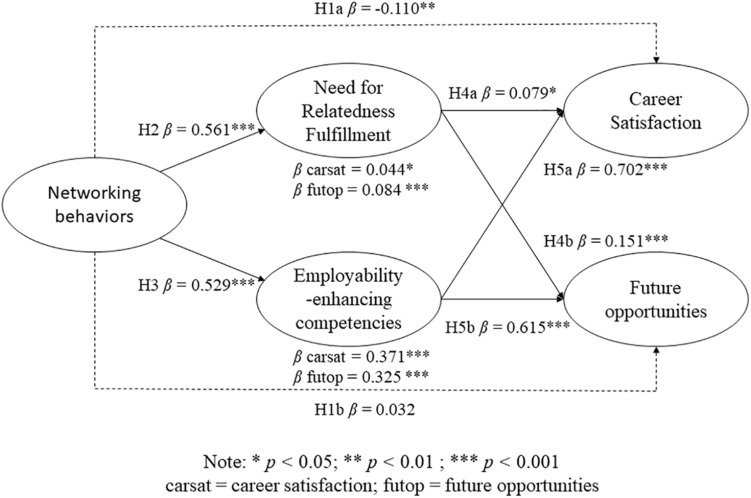
Final model.

We fitted two structural models, both showing an adequate fit. The first was a partially mediated model in which networking behaviors also loaded directly on our two dependent variables: career success operationalized as career satisfaction and perceived future career opportunities [χ^2^(185) = 1204.252, *p* < 0.001, CFI = 0.93, TLI = 0.92, RMSEA = 0.064]. The second was a fully mediated model with the direct relationship between networking behaviors and both independent variables being constrained to zero [χ^2^(187) = 1218.676, *p* < 0.001, CF I = 0.93, TLI = 0.92, RMSEA = 0.064]. The first, hence partially mediated, model provided a significantly better fit according to the Chi-square test [χ^2^(2) = 14.424, *p* < 0.001]. As such we report the findings for the partially mediated model in Table 2. Figure 2 summarizes our main findings. In what follows, we first report the findings for career satisfaction, followed by the findings for perceived future career opportunities.

**TABLE 2:** Regression coefficients of the structural model.

**Outcome variable**	**Independent variable**	**β (standardized)**
**Career satisfaction**		
	Networking behaviors (H1a)	–0.110^∗∗^
	Need for relatedness fulfillment (H4b)	0.079^∗^
	Employability-enhancing competencies (H5b)	0.702^∗∗∗^
	*Total effect networking*	0.305^∗∗∗^
	*Indirect effect networking through Need for relatedness fulfillment*	0.044^∗^
	*Indirect effect networking through employability*	0.371^∗∗∗^
	Career length	0.004^∗^
	Number of years active as an independent	0.002
	Belongs to a network (dummy-coded)	–0.061
**Future opportunities**		
	Networking behaviors (H1b)	0.032
	Need for relatedness fulfillment (H4b)	0.151^∗∗∗^
	Employability-enhancing competencies (H5b)	0.615^∗∗∗^
	*Total effect Networking*	0.442^∗∗∗^
	*Indirect effect Networking through need for relatedness fulfillment*	0.084^∗∗∗^
	*Indirect effect Networking through employability*	0.325^∗∗∗^
	Career length	–0.002
	Number of years active as an independent	–0.000
	Belongs to a network (dummy-coded)	−0.110^∗^
**Need for relatedness fulfillment**		
	Networking behaviors (H2)	0.561^∗∗∗^
	Career length	0.006^∗∗^
	Number of years active as an independent	–0.000
	Belongs to a network (dummy-coded)	0.114
**Employability-enhancing competencies**		
	Networking behaviors (H3)	0.529^∗∗∗^
	Career length	0.010^∗∗∗^
	Number of years active as an independent	–0.002
	Belongs to a network (dummy-coded)	0.112

*Model fit: CFI = 0.93, TLI = 0.92, RMSEA = 0.064, χ^2^(185) = 1204.252, *p* < 0.001. ^∗^*p* < 0.05, ^∗∗^*p* < 0.01, ^∗∗∗^*p* < 0.001.*

Surprisingly, and in contrast with Hypothesis 1a, we found a negative direct association between networking behaviors and career satisfaction (β networking = −0.110, *Z*-value = 2.970, *p* = 0.003). Networking behaviors were, however, significantly and positively associated with both need for relatedness fulfillment (β networking = 0.561, Z-value = 15.449, *p* < 0.001) and self-perceived employability-enhancing competencies (β networking = 0.529, *Z*-value = 14.683, *p* < 0.001), thereby providing support for Hypotheses 2 and 3. In turn, need for relatedness fulfillment (β relatedness = 0.079, Z-value = 2.433, *p* = 0.015) was positively related to career satisfaction, as was employability-enhancing competencies (β employability = 0.702, *Z*-value = 17.133, *p* < 0.001). This outcome thus suggests partial mediation of networking behaviors on career satisfaction through need for relatedness fulfillment and employability-enhancing competencies. Formal tests confirm this, with both a significant indirect of networking behaviors through need for relatedness fulfillment (β networking = 0.044, *Z*-value = 2.409, *p* = 0.016) and employability-enhancing competencies (β networking = 0.371, *Z*-value = 12.489, *p* < 0.001). All in all, this indicates relatively ‘weak’ support for H4a and ‘stronger’ support for H5a. Furthermore, looking at the net effect of networking behaviors on career satisfaction, this being the sum of both the direct and indirect effects, we found a positive relationship between networking behaviors and career satisfaction (β networking = 0.305, *Z*-value = 9.992, *p* < 0.001).

Networking behaviors did not have a significant direct association with the outcome of perceived future career opportunities (β networking = 0.032, *Z*-value = 0.830, *p* = 0.407), thereby disconfirming Hypothesis 1b. Both need for relatedness fulfillment and employability-enhancing competencies were, however, significantly and positively associated with perceived future career opportunities (β relatedness = 0.151, *Z*-value = 4.395, *p* < 0.001; β employability = 0.615, *Z*-value = 15.147, *p* < 0.001), herewith supporting Hypotheses 4b and 5b. There was a significant indirect association between networking behaviors and perceived future career opportunities through both need for relatedness fulfillment (β networking = 0.084, *Z*-value = 4.276, *p* < 0.001) and employability-enhancing competencies (β networking = 0.325, *Z*-value = 11.728, *p* < 0.001), suggesting a fully mediated relationship between networking behaviors and perceived future career opportunities through our hypothesized mediators. The total effect of networking behaviors was, unsurprisingly, positive (β networking = 0.442, *Z*-value = 13.245, *p* < 0.001). All in all, we found support for Hypotheses H4b and H5b.

All in all, our findings provided support for Hypotheses 2, 3, 4, and 5, whilst Hypothesis 1 received mixed support. There is evidence for a total positive effect of networking behaviors on both career satisfaction and perceived future career opportunities, but a small direct negative effect on career satisfaction.

## Discussion

In this study, we examined the importance of networking for freelancers’ careers. Specifically, we tested whether networking behaviors are positively associated with career satisfaction and perceived future career opportunities. Interestingly, and in contrast with our first hypothesis we found a negative direct association between networking behaviors and career satisfaction, whilst the direct association between networking behaviors and perceived future career opportunities was not significant. Using a sustainable career perspective (Van der Heijden and De Vos, 2015; De Vos et al., 2019) and resource management perspective (Spurk et al., 2019), we investigated whether networking behaviors may facilitate freelancers’ subjective career success via the mediating role of need for relatedness fulfillment and employability-enhancing competencies. Our findings largely support our hypotheses: networking behaviors are positively related to freelancers’ need for relatedness fulfillment (H2) and employability-enhancing competencies (H3) and these variables mediate the relationship between freelancers’ networking behaviors and subjective career success, operationalized in terms of career satisfaction (H4) and perceived future career opportunities (H5).

Our findings contribute to the careers literature and practice in this field in several ways. A first contribution of this study can be found in the role of need for relatedness fulfillment and employability-enhancing competencies as mediators in the relationship between networking behaviors and career outcomes. By studying these mediators, we add to previous scholarly work on the possible role of resource management behaviors in understanding career success (Spurk et al., 2019). In particular, our findings shed further light on the importance of need for relatedness fulfillment for optimal functioning. The fulfillment of this need might be particularly challenging for freelancers who are lacking the embeddedness within an organizational context and a team of ‘real’ colleagues who might contribute to fulfilling their need for relatedness at work. Our findings suggest that through career-related networking, freelancers generate social resources that may compensate for this and thus fulfill their socio-emotional needs (Bozionelos, 2015), which in turn has a positive association with subjective career success. Our study also points to the value of career-related networking behaviors as a specific strategy for building up employability-enhancing competencies that are vital in the light of one’s career sustainability, a perspective that has been largely neglected until now in earlier scholarly work (Bozionelos, 2015). In general, our study confirms previous studies (Akkermans and Tims, 2017; Hirschi et al., 2018) suggesting that the way in which individuals utilize their resources is critical for one’s career success and that the individual’s career potential seems to influence their subsequent career opportunities and outcomes (Van der Heijden et al., 2009b). Together these results provide further empirical validation of the idea that career development is inherently relational (Hall, 1996, 2004).

Second, to the best of our knowledge, this is the first empirical work that examines a mediation model between networking behaviors and career outcomes, taking a resource management perspective (Spurk et al., 2019). As such, it responds to the call for further theory development and strong empirical studies that advance our understanding of sustainable careers (Savickas and Porfeli, 2012; De Vos et al., 2019). Our data supports the mediation model we proposed and as such this research confirms the premise of earlier studies that in order to have a sustainable career, a sound interaction of the individual with their surrounding stakeholders is of utmost importance given the increasing complexity, both at work and in one’s private life (Wolff and Moser, 2009; De Vos et al., 2019). As such, adding to the work of Lo Presti et al. (2018), career-related networking behaviors are especially for freelancers essential for sustaining and further enhancing their career success and future career prospects. The generation of social resources, via networking, helps to fulfill the basic need for relatedness and to develop employability-enhancing competencies, thereby subscribing to the importance of careers as an inherently relational phenomenon (Hall, 1996, 2004; Yip et al., 2018).

An important implication for theory is the observation of a negative direct effect of networking behaviors on career satisfaction in the present study and the lack of a significant direct effect on perceived future career opportunities. One possible reason might be found in different aspects that are involved in the freelancers’ job itself. Freelancers have by nature relationships with multiple stakeholders that provide them with job-related tasks, for example. These relationships can be very diverse in goal and intensity. This might lead to an aversion regarding networking and as such have a negative impact on career satisfaction. An alternative explanation might be that freelancers may consult a wider range of stakeholders for career-related networking, who might provide them with very different and contrasting information which may lower their perceived career success. Our findings suggest that whilst networking in itself is not automatically beneficial, it does have positive outcomes via the mechanisms of need for relatedness fulfillment and employability-enhancing competencies. As such they support the idea that networking is a resource-generating activity that enhances beneficial outcomes through the resources that it generates. This provides a more nuanced view on the role of networking which in fact provokes to consider its outcomes from a more sustainable perspective. In fact, our findings suggest that it is not the act of networking in itself that is most critical, but that the social resources that an individual generates through the networking, are essential for generating an impact on career outcomes. This is interesting from the perspective of sustainable careers theory where it is posited that the individual should interact with its surrounding context in view of developing a sustainable career and that this social dimension of a career should not be underestimated in understanding sustainable career success (De Vos et al., 2019).

Finally, with this study we provide relevant new insights into the careers of an increasingly important group of workers in today’s labor market, i.e., freelancers. This research helps closing the knowledge gap on the careers of freelancers by building on earlier empirical research on central career concepts (career-related networking, employability-enhancing competencies, subjective career success) (e.g., van der Heijde and van der Heijden, 2006; Van der Heijden et al., 2009b, Van der Heijden et al., 2018; Olson and Shultz, 2013) and on basic psychological need fulfillment (Van den Broeck et al., 2016), using freelancer data.

### Limitations and Future Research

It is clear that our study has several key strengths, including a theoretical expansion of the importance of networking for freelancers’ career success. However, we also note the limitations of this study. The first limitation concerns our data collection. Data was collected using an online survey, and some response set consistencies or common-method bias may exist (Podsakoff et al., 2012). To overcome this limitation, we did our best to minimize common-method variance while designing the study, for example, by applying short questionnaires as recommended in procedural methods for reducing common-method bias (Podsakoff et al., 2012). A second limitation concerns the use of self-ratings. More scholarly work is needed to better understand how this might have influenced our pattern of results. Against this background, self-ratings of employability seem to have been an appropriate choice for getting a more nuanced picture in this particular study on freelancers.

To obtain a better understanding of the role of networking for freelancers’ career outcomes via the mediating role of resources, we suggest that future research could use a more fine-grained measure of networking, to assess potential differential relationships with outcomes depending on the type of networking or by further elaborating upon the different persons with whom freelancers network. Future research should thereby incorporate the utilization of resources generated by different stakeholders in freelancers’ networks, including their clients, labor market intermediaries, or other freelancers.

Finally, to further validate our results, it would be worthwhile to replicate this study in a longitudinal way. Longitudinal data would enhance our understanding of the importance of networking behaviors during different time periods in one’s career. Furthermore, future research should focus on a detailed examination of the direct relationship between networking behaviors and career success for freelancers. In doing that, cross-sectional research is needed to unravel all aspects of freelancers’ networking behaviors. Of course, the model can also be enhanced by testing for other moderating effects, for instance by taking the personality of the freelancers into account.

### Practical Implications

This study offers some guidelines for freelancers, policy-makers and other stakeholders being involved in freelancers’ careers. In the current labor market, there is a growing proportion of workers who are not employed as an employee, yet offering their services to organizations as independent workers. From a sustainable career perspective, especially in an era of rapid technological change and innovation, this provokes important questions regarding the long-term career success of this group of workers. This might not only be a concern for the individuals involved but also for policy makers. For instance, while it might be of utmost importance for freelancers to keep their competencies relevant in order to safeguard their career potential, this is considered as being entirely their responsibility and also their investment (in time and money). In the long run, when freelancers neglect investing in their employability, this might undermine their opportunities for obtaining new projects from clients – ultimately risking them to become unemployable. This contrasts with the situation for paid employees where employers might be more involved in ensuring that their workforces remains ‘future-proof.’

The same reasoning holds for need for relatedness fulfillment. There is convincing evidence that feeling connected with others is a critical social resource for all people, and for many people this connection is to a significant extent being realized through their work. Yet, feeling one belongs to a team or group is not obvious for freelancers when being only loosely coupled with other employees in an organization – if they aren’t doing a purely individual task from home. Especially given that labor legislation might withhold employers from taking any initiative to embed the freelancer in a team. When freelancers don’t take initiative to create connections and thus enable themselves to have a feeling of belongingness, they risk to feel an outsider which might dissatisfy their need for relatedness which, as our findings show, might have negative implications for their subjective career success.

Such networking behaviors don’t necessarily need to focus on the people working in the organizations where they provide their services, but can for instance be realized by actively participating in a network of freelancers – albeit some might feel reluctant to do this when they conceive other freelancers as potential competitors. Interestingly, our descriptive findings show that about one out of three (31%) freelancers mentioned that they were part of a network of freelancers and of those, 43% said this to be a network for informal exchanges whilst 37% was formally working together with other freelancers via a legal structure. So, 69% was not part of a network at all.

This brings us to the question how freelancers can be supported in enabling their own long-term career success, and thus career sustainability. It is clear that investing in developing and maintaining strong professional networking behaviors is a fulfilling strategy to enhance one’s career success. This needs to be added with building skills that enhance the employability competencies of freelancers and bring them in contact with peers. For example stimulating co-working spaces and training programs in networking skills could be beneficial. The question is of course, whose responsibility this is. Intermediary parties such as career counselors, labor market intermediaries or occupational federations might play a role here in creating awareness amongst freelancers regarding the importance of actively taking charge of their career by generating social resources via networking. Existing freelancer associations, but also the upcoming platforms stimulating the so-called gig economy might also play a role in this. In doing so, spreading the results of studies like ours might help to support this claim with evidence.

## Conclusion

In conclusion, in this study we found that freelancers’ networking behaviors were not directly and positively associated with career success: a negative direct association between networking behaviors and career satisfaction was found and there was no direct significant association between networking behaviors and perceived future career opportunities. Interestingly, and in line with our expectations, both need for relatedness fulfillment and employability-enhancing competencies mediated the relationship between networking behaviors and subjective career success. Workers and policy makers should therefore mainly focus on the fulfillment of the need for relatedness and employability-enhancing competences. Our findings suggest that, rather than the networking behavior itself, the resources that are built through the networking appear to be decisive for freelancers’ career success.

## Data Availability

The datasets generated for this study are available on request to the corresponding author.

## Ethics Statement

The studies involving human participants were reviewed and approved by the Ethics Committee of Antwerp Management School. Written informed consent for participation was not required for this study in accordance with the national legislation and the institutional requirements.

## Author Contributions

SJ, AD, and DS contributed to the design and implementation of the research, and to the analysis of the results. BH contributed to the development of the theoretical framework. All authors discussed the results and contributed to the final manuscript.

## Conflict of Interest Statement

The authors declare that the research was conducted in the absence of any commercial or financial relationships that could be construed as a potential conflict of interest.
